# Lyme carditis with complete heart block: management with an external pacemaker

**DOI:** 10.1002/ccr3.934

**Published:** 2017-04-26

**Authors:** Muhammad Ali Chaudhry, Srinivasa D. Satti, Ira R. Friedlander

**Affiliations:** ^1^ Department of Cardiology and ElectrophysiologyAultman HospitalCantonOhio

**Keywords:** Complete heart block, external pacemaker, Lyme carditis, management

## Abstract

Timely diagnosis and prompt initiation of treatment is essential in Lyme carditis to achieve favorable prognosis. Externalized permanent pacemaker with an active fixation lead as supportive pacing modality is a feasible option till complete resolution of conduction block with continued antibiotic therapy.

## Introduction

Lyme carditis is an uncommon disease entity with potential complications of the conductive system such as advanced atrioventricular (AV) nodal block which can be life‐threatening if quick diagnosis and early therapy is not initiated. Here, we present an interesting case of a 26‐year‐old gentleman with Lyme carditis who was managed with emergent external pacemaker placement and antibiotic therapy.

## Case Report

A 26‐year‐old gentleman with no significant past medical history had been on a fishing trip 3 weeks prior to presentation and reported a possible tick bite to his right second toe. This was followed by a mild erythematous rash and swelling on his toe that resolved within 2 days. Two weeks later, he started having episodic subjective fevers and chills and developed a well‐circumscribed macular rash on his left upper abdomen which gradually cleared within two to 3 days. He did not seek medical attention at any time.

**Figure 1:**
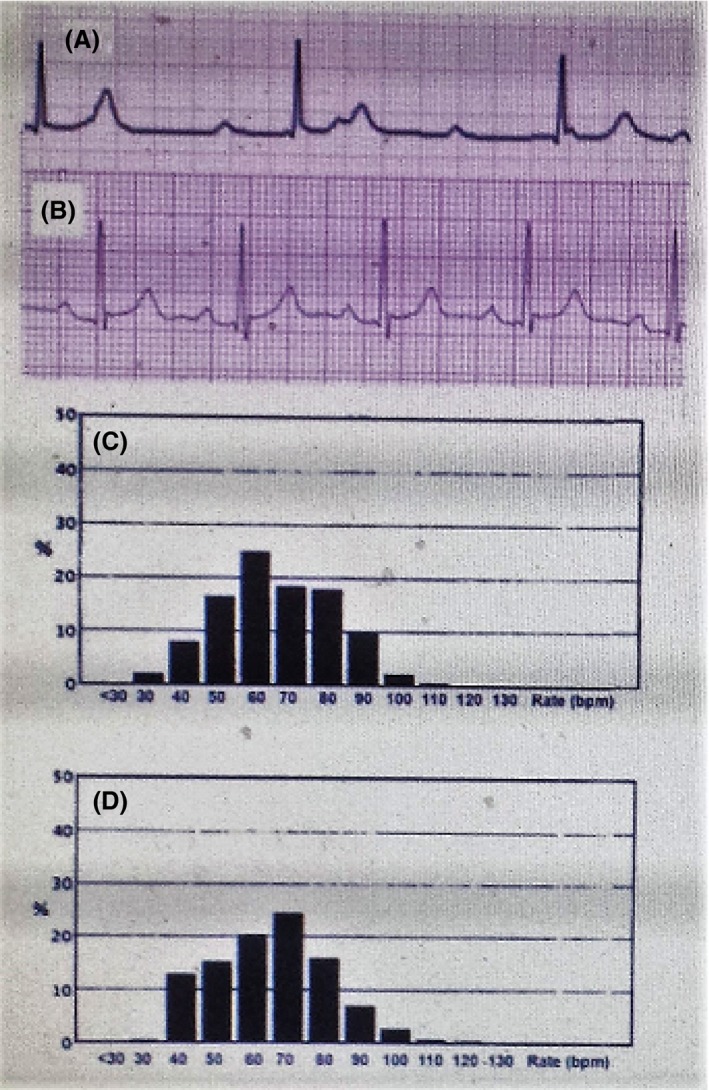
(A) Electrocardiogram (EKG) with complete heart block on initial presentation, (B) EKG with resolution of complete heart block after 3 weeks of ceftriaxone, (C) heart rate histogram with continued bradycardia at 2 weeks, (D) heart rate histogram with improved heart rate after 3 weeks of treatment with ceftriaxone.

He presented to an outside emergency room with presyncope and worsening fatigue for 1–2 days prior to presentation. Initial telemetry monitor strips demonstrated 2:1 AV block alternating with complete heart block and a junctional escape rhythm in the range of 30 beats per minute (bpm) (Fig. 1A). He was hemodynamically stable. He was started empirically on intravenous (IV) ceftriaxone therapy due to suspected Lyme disease and Ig M and Ig G Lyme titers were sent for outside analysis. A 2‐D echocardiogram demonstrated normal right and left ventricular systolic function with no significant valvular dysfunction.

**Figure 2:**
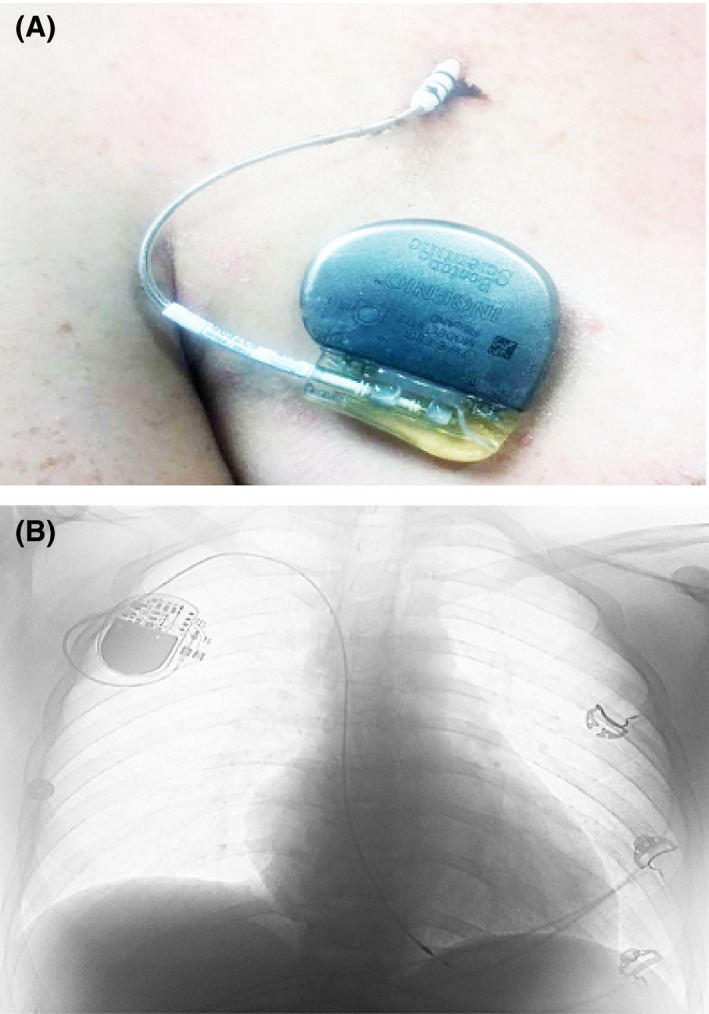
(A) Active fixation permanent pacemaker lead and externalized permanent pacemaker generator, (B) chest X‐ray in postero‐anterior projection (P‐A) with right ventricular (RV) lead positioned in apex.

Twenty‐four hours after admission, he had loss of junctional escape with underlying complete heart block and asystole with near syncope. Emergent right subclavian access was obtained for temporary pacing and placement of an active fixation permanent pacemaker lead. The lead was connected to an externalized permanent pacemaker generator as illustrated in Figure 2A with settings at VVIR‐ 60–120 bpm. Chest X‐ray confirmed appropriate lead positioning in the right ventricle as shown in Figure 2B.

Evaluation was performed to detect an underlying rhythm daily, and there was partial intrinsic recovery following 6 days of antibiotic therapy with underlying complete heart block and junctional rhythm at 30 bpm. On the ninth day of his hospital stay, the junctional rates had increased to 45 bpm and the pacemaker was programmed into a backup mode at VVI‐30. By the 12th day of admission, he was maintaining junctional rhythm with rates ranging from 45 to 50/min with periods of AV block and 2:1 AV conduction.

Initial Lyme screening with enzyme‐linked immunosorbent assay (ELISA) was positive followed by confirmatory Western Blot for Ig M. A peripherally inserted central catheter (PICC) was placed due to his previous good response to IV ceftriaxone and infectious disease recommendations for 4 weeks of IV antibiotics due to high grade AV block. He was discharged home on the twelfth day with IV ceftriaxone therapy planned for a total of 4 weeks and backup externalized pacemaker in place.

He was followed up in clinic 1 week postdischarge, and at that time on pacer interrogation, it was seen that he had not required any backup pacing and was maintaining normal sinus rhythm at 55–60 bpm. The external pacemaker was left in place. On the second follow‐up a week later, he was still maintaining normal sinus rhythm and had not required any pacing. The external pacemaker and active fixation lead were removed. An electrocardiogram (EKG) demonstrated complete resolution of AV block (Fig. 1B). Comparative heart rate histograms at two and three weeks of antibiotic therapy are shown in Figure 1C and D, respectively. The antibiotic regimen was continued to complete 4 weeks of therapy.

## Discussion

Lyme disease is a systemic illness caused by the spirochete Borrelia burgdorferi. The mode of transmission is via Ixodes scapularis tick with initial constitutional symptoms such as fever and malaise. Typically there is an annular skin rash termed erythema migrans about 5–7 cm in diameter at the site of the tick bite 1. There is a male‐to‐female predominance of 3:1 and the usual age range of patients with third degree heart block is 10–45 years 2, 3. Without timely diagnosis and effective treatment, there is widespread dissemination with neurologic, musculoskeletal, and cardiac involvement.

Lyme carditis is a dreaded complication of disseminated Lyme disease and is caused by direct cardiac invasion by spirochetes. There is initially a transmural inflammation with macrophages and neutrophils followed by a band‐like appearance of lymphocytes. Interstitial fibrosis as well as small and large vessel vasculitis, pericarditis, myocarditis, acute coronary syndromes, and coronary artery aneurysms can potentially occur 4, 5, 6.

Conduction system involvement is diverse, possibly including but not limited to bundle branch block, intraventricular conduction delay, prolonged QT interval, ventricular and fascicular tachycardias, and complete heart block 7, 8.

Atrioventricular block can develop and quickly advance from first degree block to second degree. A PQ interval of greater than 300 msec significantly increases the possibility of first degree AV block progressing rapidly to third degree AV block 9. In up to 1% of patients with Lyme disease, complete heart block can develop 10, 11. The average time from initial presentation to complete heart block is 2 weeks (2–24 days). The time for resolution of complete heart block, after treatment is initiated, is usually 6 days although it can vary from 1 day to 7 weeks 12.

Clinical features such as tick bite, erythema migrans, arthritis, and AV nodal conduction blocks typically provide strong diagnostic evidence of Lyme involvement, but primary conduction system disease without evidence of tick bite or rash can make the diagnosis challenging. Symptoms such as presyncope, syncope, worsening shortness of breath, or chest pain can be suggestive of cardiac involvement. Positive enzyme‐linked immunosorbent assay (ELISA) test for Lyme antibodies is sensitive but not specific. In the first 6–8 weeks of clinical illness, serology can be negative as well. Presence of Ig M antibodies by Western Blot analysis is usually confirmatory 13.

The usual therapeutic regimens for Lyme carditis include ceftriaxone 2 g intravenously daily for 3–4 weeks or doxycycline 100 mg twice daily or amoxicillin 500 mg three times daily for 1 month 14. Patients who are symptomatic (e.g., syncope, dyspnea, or chest pain) or those with high degree AV block should always be treated with intravenous antibiotic therapy. Transcutaneous or transvenous pacing is indicated in cases of escape rhythm or complete heart block 15.

## Conclusion

In our case, the initial symptomatology along with the skin rash occurred at 2 weeks following the tick bite. Complete heart block occurred at 3 weeks. With appropriate treatment, complete resolution was achieved within 19 days. In addition, an externalized permanent pacemaker system utilizing a transvenous active fixation permanent pacing lead placement, enabled the patient to be ambulatory, and to be discharged home with pacing support while awaiting conduction recovery on the IV antibiotics. This case highlights the importance of timely diagnosis and prompt initiation of treatment to prevent morbidity and mortality and achieve favorable prognosis in this rare progressive disease with high dissemination potential. In this clinical scenario, we have safely documented the use of an externalized permanent pacemaker with transvenous access as supportive pacing modality until complete resolution of conduction block occurred.

## Authorship

All authors have contributed to the manuscript content and have been a part of the actual case management.

## Conflict of Interest

None declared.
